# Clinical outcomes of ceramic femoral prosthesis in total knee arthroplasty: a systematic review

**DOI:** 10.1186/s13018-019-1090-4

**Published:** 2019-02-19

**Authors:** Shuai Xiang, Yan Zhao, Zeng Li, Bin Feng, Xisheng Weng

**Affiliations:** Department of Orthopaedic Surgery, Peking Union Medical College Hospital, Peking Union Medical College, Chinese Academy of Medical Science, Beijing, 100730 China

**Keywords:** Ceramics, Total knee arthroplasty, Outcomes

## Abstract

**Purpose:**

Ceramic bearings have been widely used in total hip arthroplasty (THA), which resulted in satisfactory clinical outcomes due to the excellent tribological characteristics of the implants. However, ceramic components are not commonly used in total knee arthroplasty (TKA) because of brittleness. This study aimed to gather and analyze information regarding the clinical outcomes (including survival without revision, causes of revision, functional outcome, and incidence of loosening) and reach a definitive conclusion about the use of ceramic femoral components in total knee arthroplasty.

**Methods:**

MEDLINE, EMBASE, Cochrane, and ClinicalTrials.gov databases were searched for studies that reported the clinical and/or radiological outcomes with or without survival data of ceramic TKA implants and that included more than 10 patients with a minimum of 1 year follow-up.

**Results:**

From an initial sample of 147, there were 14 studies that met the inclusion criteria. Overall, there was a notable enhancement of joint function after the procedure, with a satisfactory mid- and long-term survival of the ceramic components, which is comparable to that of the conventional alloy components reported previously. In addition, the revision rate was reported to be between 0% and 14.37% according to the included studies. However, revision due to aseptic loosening, wear, and component fracture appeared to be rare, demonstrating the safety of in vivo use of ceramic bearings in the TKA procedure.

**Conclusions:**

Ceramic TKA implants show similar post-operative clinical results and survival rate compared to their conventional metallic counterparts. Our results confirmed the safety of in vivo use of ceramic bearings in TKA, with rare implant breakage and aseptic loosening. Considering the excellent characteristics of the tribology of ceramics, the clinical use of ceramic prostheses in TKA could be promising.

## Introduction

Total knee arthroplasty (TKA) is regarded as an effective and reliable treatment to restore joint function for end-stage arthritis [1–3]. Today, cobalt-chrome-molybdenum alloy (CoCrMo) is widely used in TKA femoral components. According to several reports, over 90% CoCrMo TKA prostheses successfully survived 15-year post-operative use [4–6]. Several factors including aseptic loosening, as well as metal hypersensitivity, which affects over 10% of the population, could jeopardize long-term survival of the implants, causing failure of TKA [7, 8]. Debris generated by wear of polyethylene (PE) components could induce osteolysis, resulting in aseptic loosening of the TKA prosthesis [9]. To reduce polyethylene wear and prolong the life span of the implant, modification of the articulating surfaces was addressed and the design of the prosthesis was improved [10, 11]. The application of ceramics instead of cobalt-chromium alloys for the femoral component could be a promising approach.

Ceramic materials, including zirconia, alumina, and their compounds, have excellent characteristics of tribology and biocompatibility and have been introduced as an alternative bearing in the design of articular prostheses for decades, especially in femoral head prostheses in total hip replacement. In several wear-simulation studies, a significantly reduced amount of polyethylene wear was observed in oxidized zirconium compared with conventional cobalt chromium alloy [12]. Recent clinical studies in total hip arthroplasty (THA) also demonstrated this advantage of decreasing polyethylene wear and thus alleviating aseptic osteolysis compared with patients in a metal-PE group [13]. Furthermore, the debris produced from the metal-PE bearing surface could elicit allergenic reactions in some of the patients receiving the TKA procedure. The medical-grade ceramic prosthesis is made of a bio-inert material and has been proved to be safe in vivo, providing a promising alternative for patients allergic to metallic materials [14].

However, contrary to their wide use in THA, ceramic bearings are rarely used in TKA, although the first ceramic TKA prosthesis was designed and used in the 1980s. However, its high brittleness and potential for implant fracture in vivo restrict its application in TKA [15, 16]. Besides, because of the smooth surface of the ceramic component, the adhesive strength of the bone cement has been questioned for some time [17, 18]. In the last decade, several studies reported long-term outcomes and survival, as well as tribological statistics of ceramic knee implants, and a few of them compared the clinical and radiological outcomes between ceramic knee prostheses and metal prostheses, demonstrating similar long-term outcomes and survival between these two types of implants. These studies provided us with new and solid evidence for the clinical use of ceramics in TKA.

The purpose of this study was to systematically review the published literature regarding the use of ceramic knee implants in TKA to evaluate their clinical outcomes and long-term survival as well as to determine whether the ceramic knee prosthesis is suitable for TKA. This study should provide new evidence for surgeons, allowing them to better understand the safety and superiority of ceramic knee prostheses and to eliminate prejudice against their use for TKA.

## Materials and methods

### Search strategy

We searched MEDLINE, EMBASE, Cochrane, and ClinicalTrials.gov databases to retrieve relevant literature up to March 2017. The following terms were used for the search: total knee replacement, total knee arthroplasty, ceramic, aluminum oxide, and zirconia. The references of articles of interest were also screened for potential studies missed in the primary search. Additionally, we supplemented our search for articles in journals by hand and contacted the authors for unpublished data if necessary. All searches were limited to human studies. Only articles in English were included. If more than one article reported data from the same cohort, only the latest one was included as the identifier. If possible, this article integrated the data from different reports of that cohort.

### Inclusion criteria and exclusion criteria

1. Type of studies. Clinical trials, prospective, or retrospective (one arm or controlled) were eligible. Articles reporting in vitro studies and simulations were excluded.
2. Participants. Patients had a diagnosis of osteoarthritis or rheumatoid arthritis, and TKA was the preferred treatment. Patients who underwent TKA due to malignancy, trauma, or other causes were excluded.
3. Intervention. The majority of the studies included patients who underwent primary TKA, with the prosthesis made of ceramics. There was no restriction on the specific chemical components of the implant. If there was a control group, these patients received TKA with a metal implant.
4. Outcomes. We included articles reporting at least one of the following parameters: peri-operative measurements (including blood loss, post-operative drainage, pain, time of operation, and length of hospitalization), post-operative complications, rate and reasons for revision, survival curve (Kaplan-Meier curve) of the implant, ability to walk, range of motion of the knee joint after surgery, or other assessment of knee joint function. The observation of a radiolucent line on X-ray image was considered equivalent to loosening.

### Data extraction

Data were extracted by two investigators independently using a collection form designed by our group. Data presented only in graphs and figures were extracted whenever possible, but were included only if consensus were achieved. Data not published was acquired by contact with the original investigators and if that failed, calculated with available data. For articles that did not report a Kaplan-Meier curve, we constructed the curve according to the data available when possible.

## Results

### Search result

**Fig. 1:**
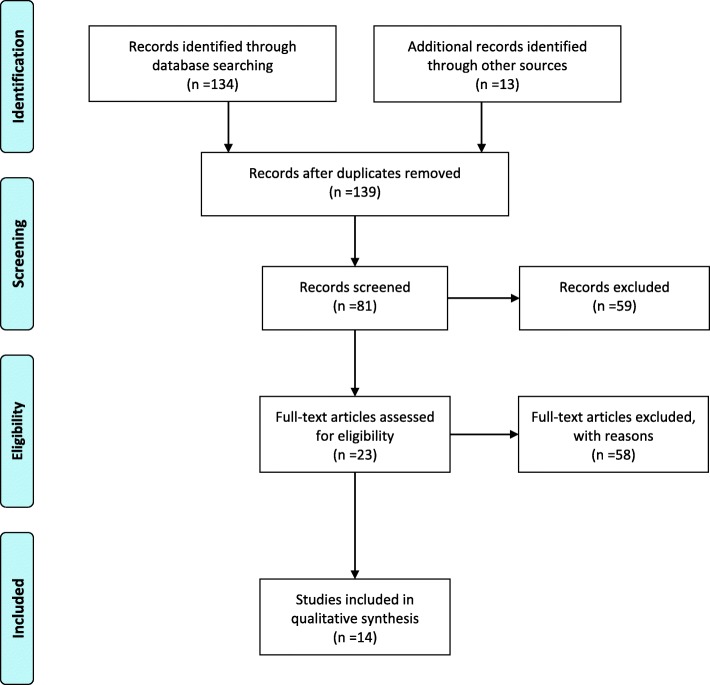
Flow diagram

A total of 147 records were retrieved, among which 134 were acquired through database searching, and 13 identified through other sources. There were 140 articles remaining after removing the duplicates, and 81 of them were screened based on the abstract, among which 23 full-text articles were further accessed for eligibility. After a thorough evaluation, 14 reports were included in this systematic review [19–32] (Fig. 1).

### Characteristics of the included studies

The characteristics of the studies included are summarized in Table 1. A ceramic femoral prosthesis was implanted in all studies. Three studies compared ceramic prostheses with metal prostheses. The duration of follow-up ranged from 1 year to more than 18 years.

**Table 1:** Characteristics of studies included

Author, year	Patients	Knees	Age at operation	Type of femur prosthesis	Type of tibial prosthesis	Duration of follow-up (years)
Akagi, 2000 [19]	136	182	68 (39–84)	Alumina	Metal	5.8 (3.9–9.0)
Koshino, 2002 [20]	64	90	59 (38–81)	Alumina	Alumina	4.67 (2.25–8.1)
Laskin, 2003 [21]	73	73	N/A	Zirconia	Metal	2
Bal, 2006 [22]	33	36	59 (29–80)	Zirconia	Metal	2.6 (2–3.3)
Majima, 2008 [23]	110	110	66 (55–77)	Alumina	Metal	5.5 (3–10.3)
Iida, 2012 [24]	80	107	72 (45–86)	Alumina	Metal	5 (1–7)
Innocenti, 2014 [25]	83	87	N/A	Zirconia	Metal	11.3 (10–12.6)
Park, 2014 [26]	67	84	55 (32–75)	Zirconia/niobium	Metal	5.16 (4.25–7.33)
Ahmed, 2015	278	303	68 (45–89)	Zirconia	Metal	9.7 (8.3–11.6)
Bergschmidt, 2015 [27]	107	109	67.8 (52–75)	Alumina/zirconia	Metal	4.08 (0–5)
Bergschmidt, 2016 [28]	21	21	66.9 (38–79)	Alumina/zirconia	Metal	5
Meier, 2016 [29]	28	32	66.87 (46–83)	Alumina/zirconia	Alumina/zirconia	1
Nakamura, 2017 [30]	51	37	72 (45–86)	Alumina	Metal	11.8 (10–13)
Nakamura, 2017 [30]	114	167	68.5 (39–91)	Alumina/yttria	Metal or alumina	18.1 (15–24.7)
Grand total	1245	1438				

Duration of follow-up was presented as a mean value with range in the brackets. *N/A* indicated the corresponding data were unavailable

### Function of knee joints

Data regarding the range of motion, the HSS score, and Knee Society knee-function (KSS) score were extracted from the included studies (Tables 2 and 3). For the evaluation of the motion of knee joints, two studies reported the range of motion, and seven studies reported the range of flexion and/or range of extension individually. For the assessment of knee joint function, the HSS scores were used in four studies, and the KSS scores were calculated in six studies. According to the original reports of the studies, the HSS and the KSS scores improved significantly after TKA with ceramic femoral implants.

**Table 2:** Function of knee joints before and after total knee arthroplasty with ceramic femoral implants

Author, year	HSS		Knee Society knee score		Knee Society function score	
	Pre-op	Post-op	Pre-op	Post-op	Pre-op	Post-op
Akagi, 2000 [19]	44.5	86.3	N/A	N/A	N/A	N/A
Koshino, 2002 [20]	N/A	N/A	41 ± 16	83 ± 14	29 ± 22	50 ± 29
Bal, 2006 [22]	N/A	N/A	39.9	92.3	42.3	69
Iida, 2012 [24]	N/A	N/A	14 ± 13	90 ± 10	47 ± 13	76 ± 22
Innocenti, 2014 [25]	N/A	N/A	36	84	37	83
Park, 2014 [26]	N/A	N/A	39 (0–70)	92 (31–100)	53 (0–90)	90 (35–100)
Bergschmidt, 2015 [27]	55.1 ± 11.5	85.6 ± 9.6	N/A	N/A	N/A	N/A
Bergschmidt, 2016 [28]	58.7 ± 12.7	88.5 ± 12.3	N/A	N/A	N/A	N/A
Meier, 2016 [29]	N/A	N/A	38.84 ± 15.75	93.66 ± 7.41	53.61 ± 18.45	95.47 ± 12.01
Nakamura, 2017 [30]	N/A	N/A	14 ± 13	89 ± 11	47 ± 13	68 ± 21
Nakamura, 2017 [30]	N/A	N/A	39.1 ± 17.9	92.8 ± 7.2	36.0 ± 19.1	47.0 ± 22.9

*HSS* Hospital for Special Surgery knee score. All data were presented as mean ± standard deviation, with range in bracket. *N/A* indicated the corresponding data were unavailable

**Table 3:** Range of motion of knee joints before and after total knee arthroplasty with ceramic femoral implants

Author, year	Flexion (degrees)		Extension (degrees)		Range of motion (degrees)	
	Pre-op	Post-op	Pre-op	Post-op	Pre-op	Post-op
Akagi, 2000 [19]	119	124	− 10.8	− 0.3	N/A	N/A
Koshino, 2002 [20]	N/A	N/A	N/A	N/A	N/A	N/A
Laskin, 2003 [21]	N/A	117	N/A	N/A	N/A	N/A
Bal, 2006 [22]	100	119	− 7	0	N/A	N/A
Majima, 2008 [23]	N/A	112 ± 17	N/A	N/A	N/A	N/A
Iida, 2012 [24]	110 ± 20	115 ± 19	− 8 ± 8	− 1 ± 3	104 ± 23	114 ± 20
Innocenti, 2014 [25]	92	118	N/A	N/A	N/A	N/A
Park, 2014 [26]	N/A	N/A	N/A	N/A	N/A	N/A
Ahmed, 2015	116 (70–140)	114 (70–130)	N/A	N/A	N/A	N/A
Bergschmidt, 2015 [27]	N/A	N/A	N/A	N/A	108.6 ± 15.4	111.4 ± 15.1
Bergschmidt, 2016 [28]	N/A	N/A	N/A	N/A	111.3 ± 18.0	121.1 ± 29.6
Meier, 2016 [29]	N/A	N/A	N/A	N/A	N/A	N/A
Nakamura, 2017 [30]	110 ± 20	117 ± 22	− 8 ± 8	− 1 ± 4	N/A	N/A
Nakamura, 2017 [30]	118.1 ± 19.8	123.7 ± 19.0	− 10.7 ± 10.7	− 1.6 ± 4.9	N/A	N/A

All data were presented as mean ± standard deviation, with range in bracket. *N/A* indicated the corresponding data were unavailable

### Revision and loosening

**Fig. 2:**
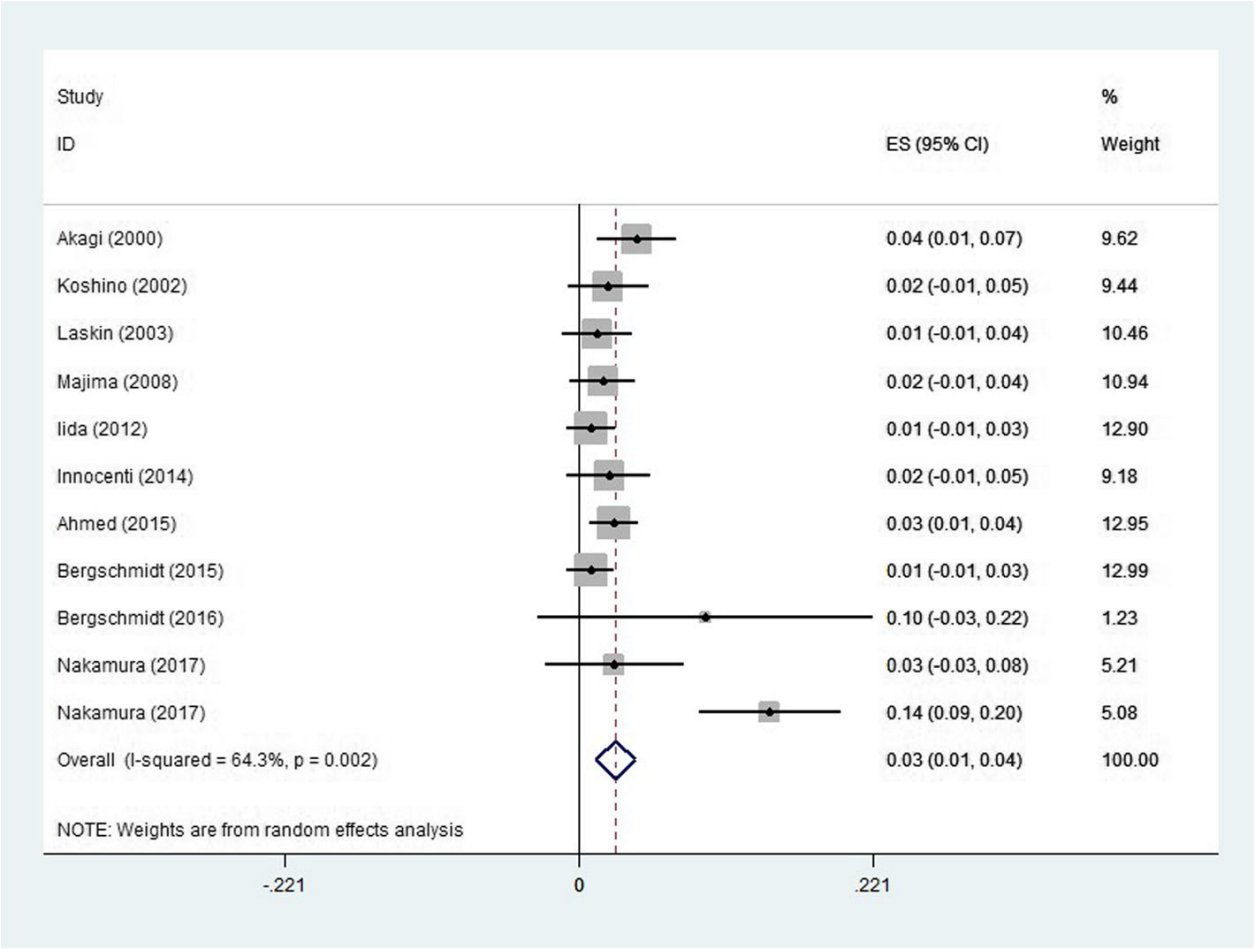
Rate of revision

The rate and detailed causes of revision and loosening are summarized in Table 4. Major causes of revision included breakage of the prosthesis, infection, aseptic loosening, instability, and wear of the prosthesis. Generally, the rate of revision increased as the duration of follow-up increased. Wear of the prosthesis, a common concern for ceramic prostheses, only occurred in three knees after following up for 18.1 years. Breakage of the prosthesis, another major concern for ceramic implants, occurred in 3 patients after a minimum follow-up of 5 years. The data were pooled to generate an overall estimate of the rate of revision, which was 0.03 (95% confidential interval 0.01 to 0.04, *I*^2^ = 64.3%, *P* = 0.002) (Fig. 2).

**Table 4:** Revision and radiolucent lines after total knee arthroplasty with ceramic femoral prosthesis

Author, year		Duration of follow-up (years)	Revision								Radiolucent line		
	No. of knees		Breakage of prosthesis	Infection	Aseptic loosening	Instability	Wear	Others	Total	Rate of revision in total	Femoral	Tibial	Patellar
Akagi, 2000 [19]	182	5.8	1	5	0	2	0	0	8	4.40%	0	0	0
Koshino, 2002 [20]	90	4.67	0	1	0	0	0	1	2	2.22%	0	0	3
Laskin, 2003 [21]	73	2	0	0	0	0	0	1	1	1.37%	0	2	0
Bal, 2006 [22]	36	2.6	0	0	0	0	0	0	0	0.00%	0	0	0
Majima, 2008 [23]	110	5.5	1	0	0	0	0	1	2	1.82%	0	3	0
Iida, 2012 [24]	107	5 (1–7)	0	0	0	0	0	1	1	0.93%	0	0	0
Innocenti, 2014 [25]	87	11.3	0	0	2	0	0	0	2	2.30%	2	12	0
Park, 2014 [26]	84	5.16 (4.25–7.33)	0	0	0	0	0	0	0	0	1	17	0
Ahmed, 2015	303	9.7 (8.3–11.6)	8 in total							2.64%	18 in total		
Bergschmidt, 2015 [27]	109	4.08	0	0	0	0	0	1	1	0.92%	4	9	0
Bergschmidt, 2016 [28]	21	5	0	1	0	1	0	0	2	9.52%	2	4	0
Meier, 2016 [29]	32	1	0	0	0	0	0	0	0	0.00%	1	5	0
Nakamura, 2017 [30]	37	11.8	0	0	0	0	0	1	1	2.70%	13	17	0
Nakamura, 2017 [30]	167	18.1	1	9	3	4	3	4	24	14.37%	44	3	0
Grand total	1438		3	16	5	7	3	10	52	3.62%	67	72	3

Duration of follow-up was presented as a mean value with range in the brackets

The adhesion between the prosthesis and adjacent tissue was evaluated by X-ray, and radiolucent lines were considered indicators for prosthetic loosening. The appearance of radiolucent lines around the femoral component, tibial component, and the patella is summarized individually in Table 4.

### Survival curve of ceramic prostheses

**Fig. 3:**
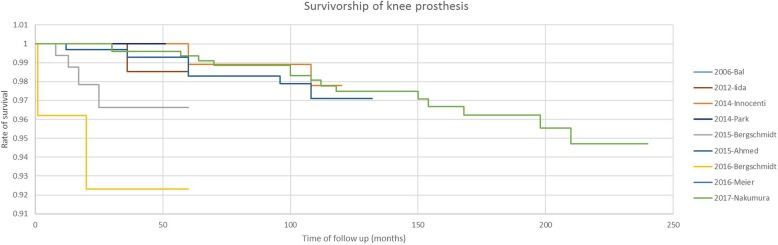
Kaplan-Meier curve of ceramic prosthesis

**Fig. 4:**
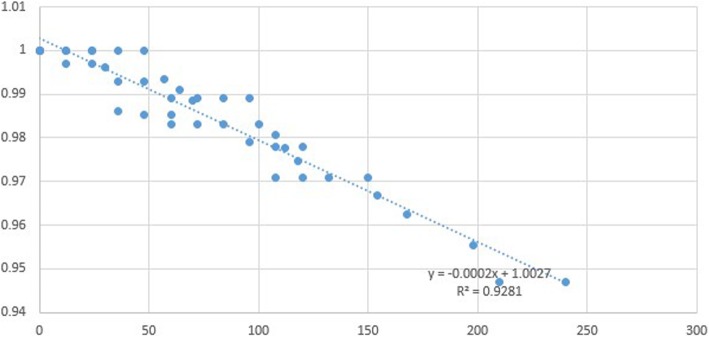
Scatter diaphragm of survival rate and time

The survival of ceramic prostheses is illustrated by a Kaplan-Meier curve as shown in Fig. 3. Four studies reported excellent survival of ceramic prostheses at follow-up, which was reflected by a survival rate of 98% 5 years after the operation and 95% 20 years after the operation. However, Bergschmidt reported a lower survival rate after the arthroplasty, with a much shorter survival period. The possible explanation was that several patients underwent unexpected events shortly after discharge (involving trauma), and needed revision for infection of the prosthesis. Additionally, we plotted a scatter diagram of the survival rate and duration based on the information from the studies included and estimated the survival-time relationship by linear approximation (Fig. 4). The function is as follows:

$$ \mathrm{Rate}\ \mathrm{of}\ \mathrm{survival}=-0.0002\times \mathrm{time}\ \left(\mathrm{months}\right)\ \mathrm{after}\ \mathrm{operation}+1.0027 $$

## Discussion

### Key findings

Given the uncertainty regarding the clinical performance of ceramic TKA components, we aimed to explore whether differences exist between the conventionally used alloy components and ceramic components. By summarizing the previous reports on ceramic TKA prostheses, in this study, we sought to systematically evaluate the clinical results, radiological results, and the survival of the ceramic prostheses. Our results demonstrated a marked enhancement of joint function after the procedure, with a satisfactory mid- and long-term survival of the ceramic components, which is comparable to that of the conventional alloy components reported previously. Furthermore, only a small fraction of revision procedures were performed due to polyethylene wear and aseptic loosening of the prosthesis, although radiolucent lines were observed in several studies. To the best of our knowledge, this is the first systematic evaluation of the results and safety of ceramic TKA components.

### Advantages and concerns of ceramic components

Today, cobalt-chromium alloy is the most widely used prosthetic material in the femoral components of total knee prostheses, showing good recovery of joint function post-operatively. Long-term follow-up has demonstrated excellent survival of the conventional cobalt-chromium prosthesis [2, 4, 5]. However, according to these reports, the debris produced by wear of the polyethylene tibial insert has agglomerated in vivo and thus induced aseptic loosening of the components, and this appears to be one of the major reasons leading to failure of total knee arthroplasty [33]. Researchers have shown that accumulation of wear particles activates macrophages and induces the release of inflammatory cytokines, which can be detected in the synovial fluid of failed TKAs [34]. These inflammatory cytokines are considered to play the pivotal part in periprosthetic bone resorption and implant loosening. Accordingly, a fundamental improvement would be to reduce the wear of the polyethylene insert and thereby decrease the occurrence of periprosthetic osteolysis and loosening of the components. In addition to improving the wear resistance of polyethylene by increasing the crosslinking, an alternative material with a more conforming surface and increased resistance to friction is needed for the articulating surface in TKA components.

With a proven superior surface conformity, high bending strength, and increased wear resistance, ceramic bearings (which are made of alumina and zirconia), have been used in THA components for many years [35]. Several in vivo and in vitro studies have also documented the superiority of ceramics in wear resistance when articulating against polyethylene compared with metal components [36, 37]. In recent years, several studies concerning the tribological statistics of ceramic knee implants have been performed and satisfactory results have been reported. Knee simulator studies held in various laboratories have also demonstrated the wear advantages of ceramic components over their alloy counterparts. The results from work by Ezzet and Spector showed that compared to cobalt-chromium, the wear rate of polyethylene against oxidized zirconium was reduced by nearly 50% after 5 million cycles of testing [38]. Another study conducted by White et al. found that after 2 million cycles of testing, the condition of polyethylene inserts articulating against oxidized zirconium was far better than against cobalt-chromium. There was also an improved surface texture for the oxidized zirconium [39]. Considering that in vitro simulator tests could not fully represent the clinical performance of the TKA components, retrieval studies were also carried out to evaluate the in vivo performance of the ceramic components. The results from Oonishi et al. showed a large difference in the wear rate for the polyethylene inserts articulating against cobalt-chromium femoral components, and the estimated linear-wear rate was 0.021 to 0.074 mm/year, while the wear rate in the group with alumina ceramic femoral components was low and stable, and was estimated as 0.026 mm/year. Furthermore, anterior-posterior-oriented scratches were found on the surface of both cobalt-chromium femoral components and polyethylene inserts using SEM, resulting from the flexion-extension movement of the joint. However, no scratching was found on the surface of the polyethylene inserts in the group with ceramic components, and the surface texture of the alumina femoral components remained unchanged [40].

In addition to having excellent wear resistance, the ceramic material is biologically inert, which is another advantage compared with the use of cobalt-chromium prostheses. Approximately, 15% of the population have been reported as being hypersensitive to nickel, usually in association with a cross-reactivity to cobalt [8]. In general, at least 1% nickel was included in the conventional cobalt-chromium femoral components, whereas there was no detectable level of nickel involved in the alumina and zirconia counterpart [41]. Moreover, the lower level of biological response to ceramic particles in vivo, represented by lower levels of tumor necrosis factor-alpha (TNF-α) and prostaglandin E2 (PGE2), makes ceramic components a promising solution for those patients with allergy to metal ions.

Two major concerns of in vivo failure of the ceramic components are fracture of the implant due to its brittleness and decreased adhesive strength of the bone cement due to the conformed surface of the ceramic components [42]. In a 2-year follow-up study for ceramic-on-ceramic THA, Hamilton et al. reported 2 intraoperative ceramic liner fractures and 2 post-operative ceramic liners chipping, from a total of 177 THAs, and the overall rate of ceramic liner-related events was 2.2% [42]. In addition, in a mechanical study concerning the strength of adhesion of the cement, the maximum adhesive strength to ceramic femoral components was substantially lower than to the Co-Cr alloy counterpart under moist conditions (*P* = 0.0017) [18]. Furthermore, in an 18.5-year follow-up study concerning cemented alumina THA, Hamadouche et al. reported the debonding rate of the acetabular cup from the bone cement was 22% (19 out of 85 hips), leading to a sudden pain and a requirement for revision of the THA [37]. This high debonding rate exacerbated concern for the adhesive strength of ceramic components.

### Clinical outcomes of ceramic TKA components

The procedure of TKA has been proven for the treatment of end-stage degenerative joint diseases. Among the included studies, a variety of evaluation parameters including range of motion, the HSS scores, and the KSS scores were used to assess the pre-operative and post-operative joint function. Overall, the post-operative joint function results were reported to be significantly improved, with an increased range of motion, greater range of flexion, enhanced HSS scores, and KSS scores in both short- and long-term follow-ups [19–23, 25, 27–32]. In 3 studies with a 10-year or more follow-up, the KSS score was improved to 83–92, with a function score of 47–84 at the last visit. The range of flexion at the last visit was also significantly improved, with a range of 118–123.7° [25, 30, 31]. In the past, there has been a lack of evidence for the long-term durability and clinical safety of ceramic TKA which limited the clinical use of the ceramic components. Recently, Nakamura reported a minimum follow-up of 15 years on ceramic tri-condylar knee implants (revision for any surgery or radiographic failure was the end point), Kaplan-Meier survival at 15 years was 94.0% (95%CI 91.4–96.5%). Other research on short- and mid-term survival of ceramic components also demonstrated satisfactory results, with a 5-year survival of 92–100% and a 10-year survival of 97–98%. In this study, we further systematically estimated the survival-time relationship by linear approximation, which is shown in Fig. 2. Among all these included studies, the revision rate was 0–14.37%. However, revision due to aseptic loosening, wear, and component fracture appears to be rare. The two most common reasons for a revision procedure are infection and fracture caused by post-operative trauma. These results demonstrated the clinical safety of ceramic TKA prostheses.

In 2014, Innocenti et al. summarized the long-term follow-up of Cr-Co femoral components using clinical scores and 10-year survival [25]. Although the post-operative range of motion was not reported, the results showed that the KSS score at 10 years was 83–96, while the function score was 74–83, with a 10-year survival over 91%. Compared to the post-operative KSS score of Cr-Co components, the KSS of the ceramic components was comparable, while the function score was lower. Because the lower function scores of the ceramic components were reported in the Japanese population (47 and 68, respectively), this discrepancy could be attributed to different recovery plans and daily activities in different populations, as well as possible bias during follow-up. Furthermore, we demonstrated ceramic TKA implants to be reliable, with a comparable 10-year survival with their alloy counterparts.

### Randomized clinical trials on ceramic TKA bearings

In the past few decades, the use of oxidized zirconium femoral components has increased worldwide. In our literature search, we also took note of three prospective, randomized controlled studies focusing on the clinical outcomes and mid-term survival between procedures using oxidized zirconium femoral components and conventional Co-Cr implants [21, 43, 44]. In a 2-year follow-up, Laskin et al. reported no significant differences in KSS score and passive flexion range between the ceramic component group and the Co-Cr group, while patients in the ceramic group showed a statistically significant increase in the rapidity of regaining flexion [21]. Similarly, two mid-term follow-ups of these randomized clinical trials showed no significant differences in clinical, subjective, radiological, and survival measurements between these two groups [43, 44]. Noticeably, in research conducted by Kim et al., the characteristics of the aspirated wear particles, including size, weight, and surface roughness were compared between the two groups, and no marked differences were found up to 7.5 years post-operatively [44]. However, these studies were not eligible for this review due to the lack of data of interest; hence, we excluded them in our review.

### Limitations

By systematically reviewing these single-armed studies, we found that ceramic components could be used in the TKA procedure, with excellent long-term joint function and survival. However, because of the limited use of ceramic TKA components worldwide, RCTs and cohort studies comparing the long-term clinical results and survival between ceramic TKA components and conventional cobalt-chromium prostheses were not available. This may jeopardize the strength of this conclusion. More research on ceramic TKA components, especially comparative studies with a higher level of evidence, are needed to support the use of ceramic components in the TKA procedure.

## Conclusion

Despite the limited use of ceramic knee implants worldwide, the KSS and survival rate of the ceramic femoral components in our study were comparable with those of the conventional metallic femoral components, while implant fractures rarely occurred. Considering the advanced tribological features of the ceramic components, we believe that these components could have a promising role in knee implant surgery.

## Abbreviations

CoCrMo: Cobalt-chrome-molybdenum
THA: Total hip arthroplasty
TKA: Total knee arthroplasty
